# Experience of financial hardship and depression: a longitudinal population-based multi-state analysis

**DOI:** 10.1017/S2045796025100115

**Published:** 2025-07-01

**Authors:** Gustave Maffre Maviel, Alexandra Rouquette, Camille Davisse-Paturet, Arthur Descarpentry, Arnaud Sapin, Nathalie Bajos, Jean-Baptiste Hazo, Anne Pastorello, Josiane Warszawski, M. Melchior, Cecile Vuillermoz, the EpiCov Study Group

**Affiliations:** 1 Social Epidemiology, Mental Health and Addictions (ESSMA), Institut Pierre Louis d’Épidémiologie et de Santé Publique (IPLESP), Paris, France; 2 Centre de Recherche en Epidémiologie et Santé des Populations, Institut National de la Santé et de la Recherche Médicale, Université Paris-Saclay, Université de Versailles Saint-Quentin-en-Yvelines, Villejuif, Paris, France; 3 Department of Epidemiology and Public Health, APHP, Paris-Saclay University, Le Kremlin-Bicêtre, France; 4 Institut de Recherche Interdisciplinaire sur les Enjeux Sociaux (IRIS), Institut National de la Santé et de la Recherche Médicale (INSERM)/École des Hautes Études en Sciences Sociales (EHESS), Aubervilliers, France; 5 Statistics Direction of the French Ministry of Health and Solidarity (DREES), Paris, France.

**Keywords:** depression, financial hardship, multi-state models

## Abstract

**Aims:**

Little is known about the effects of both financial hardship and people’s perception of it on mental health. This study aimed to evaluate the effect of perceived financial hardship on individuals’ depressive symptoms across several strata of objective financial situations.

**Methods:**

We used data from a four-wave French national population-based cohort (*N* = 14,236, 2020–2022) to assess the relationship between depressive symptoms and perceived financial hardship. Multi-state models (MSM) were used on a three-level scale for depressive symptom severity based on the Patient Health Questionnaire (PHQ-9). Analyses were stratified by household income to study the interaction with the objective financial situation.

**Results:**

We showed a link between perceived financial hardship and the onset and deterioration of depressive symptoms in subsequent waves, with effect sizes ranging from HR = 1.29 (0.87-1.90) to 2.23 (1.66-2.98). This association was stronger in the high-income population. There was no significant link between perceived financial hardship and the improvement of depressive symptomatology.

**Conclusions:**

This study confirms that perceived financial hardship is linked to the onset and deterioration of depressive symptoms. Furthermore, it suggests a stronger effect in high-income households, which could mean that the experience of financial hardship and the objective financial situation interact in their effect on mental health.

## Key messages

- Our study showed an association between perceived financial hardship and subsequent depressive symptoms in a four-wave French national cohort.
- This association seemed stronger among people belonging to higher-income strata.
- Multi-state models offer an original perspective on the evolution of depressive symptoms, corresponding to theoretical models of depression.

## Introduction

Depression and symptoms of depression have consistently been associated with various social determinants (educational level, household income, matrimonial status, etc.) (Assari, 2017; Ettman *et al.*, 2022; Lorant *et al.*, 2003), including financial hardship, which is measured by the extent to which households lack essential material and immaterial resources (Butterworth *et al.*, 2009; Heflin and Iceland, 2009). It was recently found to be a stronger predictor of depression than several other socioeconomic variables, including educational level and unemployment (Butterworth *et al.*, 2012). It has also been suggested that individuals’ perception of their financial circumstances could be an underlying predictor of depression, moderating the effect of objective financial hardship, although the results remain unclear (Asebedo and Wilmarth, 2017; Dunn *et al.*, 2008). This hypothesis is in line with studies exploring the link between financial stress and depression rather than objective financial hardship (Guan *et al.*, 2022; Sekścińska *et al.*, 2022). However, there is currently a lack of research evaluating the combined effects of both objective financial hardship and the perception of hardship on depression. The association could also be bidirectional, as depressive disorders could induce financial hardship as well as a negative perception of it (Curran *et al.*, 2021). Furthermore, most previous studies have used a dichotomous indicator for depression, which could fail to reflect the diversity of depressive symptoms' severity.

The COVID-19 pandemic shed light on mental health and socioeconomic issues, leading to increased research on these subjects. The prevalence of depression and other mental health disorders spiked in 2020 compared to the pre-pandemic period in many countries around the world (Cénat *et al.*, 2021; Morin *et al.*, 2021). Following the same pattern, financial stressors such as job uncertainty became more predominant throughout the pandemic (Brewer and Gardiner, 2020; Godinić *et al.*, 2020). In the last couple of years, both of these evolutions have paved the road for further research into mental health and financial stressors. France was not spared by this phenomenon, with a significant increase in the prevalence of depressive symptoms (Hajek *et al.*, 2022; Ramiz *et al.*, 2021). Few studies, and even fewer longitudinal studies, have explored the relationship between financial hardship and depression during the COVID-19 period. The same holds true when looking for studies evaluating the effect of the perception of financial difficulties on mental health. Study of this subject could be helpful to steer public interventions in mental health and financial well-being, including guidance as to whether public health or economic measures are required. One of the key issues for further surveillance and a better understanding of the underlying mechanisms is the relevance of different measures of socioeconomic position, and specifically the relative importance of subjective vs. objective measures. In order to address this lack of evidence, our aim was to estimate the effect of perceived financial difficulties on depressive states, also taking into account individuals’ objective financial situation using data from the French EpiCov cohort, a random national population-based study that took place during the COVID-19 pandemic.

A variety of statistical methods could be used to explore the link between perceived financial hardship and depressive symptoms. Most longitudinal studies on this topic have opted for mixed-effects regression models or trajectory modelling (Curran *et al.*, 2022; Pan *et al.*, 2012; Zheng and Jia, 2022). However, these models often consider depression as a linear or binary outcome, putting aside the temporal evolution of the symptoms (e.g., onset, deterioration, improvement). Multi-state models (MSM) offer a dynamic state-based approach, which provides answers to this issue. These models have been used in recent studies centred on mental health outcomes, including depression (Keown-Stoneman *et al.*, 2015; Meyer *et al.*, 2019; Xiong *et al.*, 2021). Their structure enables distinct effects to be detected across different states of depression while also making it possible to control for adjustment variables. To be statistically efficient, MSM require a large number of transitions across depressive states. This requirement was met during the pandemic, given the increases in both financial hardship and mental health disorders over that period(Dragano *et al.*, 2022; Sekścińska *et al.*, 2022). MSM have the advantage of using interpretable hazard ratios, which clearly indicate the risk of onset and deterioration of depressive symptoms. MSM expand their interpretability further by supplying distinct effects for each type of transition. On the other hand, regression models using psychometric scales account for the effect of covariates on the severity of symptoms. Therefore, MSM offer an interesting alternative to predict the transitions from one depressive state to another.

Thus, the main aim of this study was to explore the effect of perceived financial difficulties on depression, taking into account the objective financial situation, using multi-state models.

## Method

### Study population and design

The EpiCov study (ORCHESTRA collaboration) is a national population-based cohort that includes participants aged 15 years and over in 2020, randomly selected from the national tax register (FIDELI) and followed from 2020 to 2022 (Warszawski *et al.*, 2022). Data collection occurred in four waves over a two-and-a-half-year period (May–June 2020, October–December 2020, June–August 2021, September–December 2022 (Davisse-Paturet *et al.*, 2023)), providing a range of information on the social determinants of health and living conditions. The sample included inhabitants from mainland France and three overseas territories (Martinique, Guadeloupe, and Reunion) and excluded residents in care homes and prisons. Data were collected using computer-assisted web interviews (CAWIs) and computer-assisted telephone interviews (CATIs). In the first wave, among the 371,000 individuals initially selected, 134,391 actually completed the questionnaire. Additional baseline socio-demographic information was provided by the national tax register (FIDELI) 2018 database.

In the first two survey waves, a 10% subsample was randomly selected to complete a longer version of the questionnaire, which included data on mental health. We included the 14,236 participants who answered this longer questionnaire at baseline.

### Measures

#### Perceived financial hardship

Perceived financial hardship was measured with the following question: “Financially, in your current household, would you say…”, with possible responses: “I am comfortable” (1), “I am alright” (2), “The situation is tight, I have to be careful” (3), “I barely make ends meet” (4), and “I cannot make ends meet without getting into debt” (5). This scale is not statistically validated but is similar to other items used in previous literature (Butterworth *et al.*, 2012; Choi *et al.*, 2023). To ensure sufficient sample size, responses were grouped into three categories on the basis of their proximity: no perceived financial hardship (1–2), moderate perceived financial hardship (3), and severe perceived financial hardship (4–5).

#### Depression

Depressive symptoms were self-reported by participants using the 9-item Patient Health Questionnaire (PHQ-9). The PHQ-9 evaluates depressive symptom severity with scores ranging from 0 to 27. It has been proven to be valid as a continuous depression severity measure (Kroenke *et al.*, 2001). The PHQ-9 also produces almost identical results whether self-reported or administered by a mental health professional (Kroenke *et al.*, 2001). To compare the two models in this study, PHQ-9 scores were divided into three categories based on an algorithm using diagnostic criteria (see section *Description of the PHQ and PHQ-9* (Kroenke *et al.*, 2001): no or mild depressive symptoms, moderate depressive symptoms (2 to 4 symptoms present at least “more than half the days” in the past 2 weeks, with one of the symptoms being depressed mood or anhedonia), and major depressive symptoms (5 + symptoms present at least “more than half the days” in the past 2 weeks, with one of the symptoms being depressed mood or anhedonia).

#### Covariates

Potential confounders were identified from the literature (Asebedo and Wilmarth, 2017; Butterworth *et al.*, 2012, 2009; Sultana *et al.*, 2021) and analyses of the survey data: household income (baseline, FIDELI 2018 database), age, higher education diploma, sex, immigration status, receipt of benefits, history of psychiatric conditions, physical disability, history of somatic conditions, household composition (baseline, first survey wave); self-reported health status, employment status, and tobacco consumption (reported at each wave). Categorical covariate coding is detailed in Table 1.

**Table 1.** Characteristics of the study population, EpiCov cohort, at baseline/wave 1 (May 2020)

Characteristic	*N* = 14,236
**Demographic characteristics**	
Age, Mean (SD)	47.9 (18.2)
Female, *n* (%)	7,667 (53.9)
Immigration status, *n* (%)	
Born FR. From FR. Parents	11,189 (81.5)
Descendant of immigrant	1,385 (10.1)
Immigrant	1,160 (8.4)
Missing	502
Type of household, *n* (%)	
Alone	2,021 (14.2)
Couple without children	3,979 (28.0)
Couple with at least one child.	5,833 (41.0)
Single-parent	1,185 (8.3)
Other	1,201 (8.4)
Missing	17
**Measures of socio-economic position**	
Household income, n (%)	
Low (1st−2nd decile)	2,420 (17.5)
Medium (3rd−7th decile)	6,161 (44.7)
High (8th−10th decile)	5,208 (37.8)
Missing	447
Employment status, *n* (%)	
Unemployed/Retired	6,898 (48.5)
Employed	7,337 (51.5)
Missing	1
Highest degree, *n* (%)	
No high school degree	2,622 (18.4)
High school degree	7,689 (54.0)
> 2 years higher education	3,925 (27.6)
Receipt of benefits, *n (%)*	2,947 (20.7)
**Health characteristics**	
History of a psychiatric condition, *n* (%)	82 (0.6)
Missing	1
Self-reported health status, n (%)	
Very Bad/Bad	517 (3.6%)
Good	13,696 (96.4%)
Missing	23
Physical disability, n (%)	463 (3.3%)
Missing	1
History of somatic condition, *n* (%)	3,733 (26.2)
Missing	1
Tobacco consumption, *n* (%)	
None	11,231 (78.9)
Less than once a day	529 (3.7)
At least once a day	2,474 (17.4)
Missing	2

### Statistical analyses

Descriptive statistics were used to describe the sample at baseline. Sampling weights (Davisse-Paturet *et al.*, ) were used to ensure national representativity. All statistical analyses were conducted using R (version 4.2.3).

Potential confounders were analysed cross-sectionally to determine their association with both perceived financial hardship and depressive symptoms. Potential confounders were selected as covariates if they had a significant cross-sectional association with both exposition and outcome in univariate and multivariate analyses (90% significance level). This first round of selection was done to exclude variables that could induce over-adjustment. No covariate from the original list was excluded.

Results from multivariate analyses were also reported, stratifying on three levels of income (1st–2nd deciles, 3rd–7th deciles, 8th–10th deciles; first level including all people living under the poverty line, second level designed to reflect middle-class income).

#### Multi-state model

**Figure 1.**
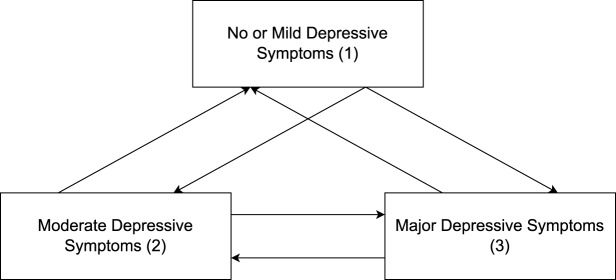
States of transition in the multi-state model.

A discrete-time multi-state model (MSM) was used to describe transitions between depressive symptom severity states across four time points during the course of follow-up. All transitions between states were allowed between each time point, but same-state transitions were not estimated (Fig. 1). The between-state transitions were similar to those of previous work involving MSM (Keown-Stoneman *et al.*, 2015; Meyer *et al.*, 2019; Zheng *et al.*, 2022).

Transitions were assumed to follow Cox proportional hazards models; each transition had a unique baseline hazard function.

Perceived financial hardship was introduced as a categorical covariate, with each level having a distinct effect on each transition. These effects were captured by hazard ratios (HR). HRs represent the proportional increase in the baseline hazard function for each perceived financial hardship level relative to the reference level (i.e., no perceived financial hardship). Covariates were all included in the model after checking that their *P*-value was less than 0.10. Analyses were conducted using the R packages *msm* (version 1.7) and *mstate* (version 0.3.2).

#### Missing data

The proportion of participants who filled out the PHQ-9 gradually decreased as the survey waves progressed, as shown in Table 2. Considering current research on multiple imputation (Hughes *et al.*, 2019; Kontopantelis *et al.*, 2017), we decided to test four different methods to handle missing data based on previous research (Supplementary Material). Reported results were computed using the third method (multiple imputation of covariates, exposure, and outcome), with other methods being discussed as sensitivity analyses. Imputation models included all covariates, exposition factor, outcome (when relevant), and sampling weights. Sixty imputed datasets (Graham *et al.*, 2007) were obtained through multiple imputation by chained equations (MICE) using the R package *mice* (3.15.0).

**Table 2.** Depressive symptoms and perceived financial hardship at each wave, epicov cohort (2020–2022)

Characteristic	Wave 1	Wave 2	Wave 3	Wave 4
Depressive symptoms severity, *n* (%)				
None or Mild (1)	12,135 (87.7)	9,684 (90.0)	7,797 (91.7)	6,745 (92.0)
Moderate (2)	1,035 (7.5)	596 (5.5)	349 (4.1)	258 (3.5)
Major (3)	664 (4.8)	486 (4.5)	354 (4.2)	327 (4.5)
Missing	402	3,470	5,736	6,906
Perceived financial hardship, *n* (%)				
None	8,210 (57.8)	6,954 (61.9)	5,728 (66.5)	4,238 (57.2)
Moderate	4,405 (31.0)	3,175 (28.2)	2,176 (25.3)	2,393 (32.2)
Severe	1,592 (11.2)	1,115 (9.9)	710 (8.2)	784 (10.6)
Missing	29	2,992	5,622	6,821

## Results

At baseline, the participants were, on average, 47.9 years old (SD = 18.2), and 54% (*N* = 7,667) were female (Table 1). Out of 14,236 participants, a total of 3.6% perceived their health status as ‘bad’ or ‘very bad’. The complete descriptive statistics, stratified by reported financial hardship, are available in the Supplementary Materials.

Table 2 shows the prevalence of depressive symptoms and perceived financial hardship across the four study waves. The prevalence of major depressive symptoms remained stable over time (4.8%, 4.5%, 4.2%, 4.5%), while levels of moderate symptoms decreased (7.5%, 5.5%, 4.1%, 3.5%). Perceived financial hardship showed no consistent trend across the study waves (31.0%, 28.2%, 25.3%, 32.2% for moderate hardship; 11.2%, 9.9%, 8.2%, 10.6% for severe hardship).

As shown in Table 3, both moderate and severe financial hardship decreased across the low, medium, and high income strata (40.5%, 38.2%, 17.8% for moderate hardship; 27.0%, 11.8%, 3% for severe hardship).

**Table 3.** Contingency table of perceived financial hardship by income strata at the wave (2020), EpiCov cohort

Characteristic	Low income (*N* = 2,420)	Medium income (*N* = 6,161)	High income (*N* = 5,208)
Perceived financial hardship, *n* (%)			
No financial hardship	783 (32.4)	3,075 (50.0)	4,121 (79.3)
Moderate financial hardship	978 (40.5)	2,347 (38.2)	928 (17.8)
Severe financial hardship	652 (27.0)	727 (11.8)	150 (2.9)
Missing	402	3,470	5,736

### Multi-state model

As shown in Table 4, perceived financial hardship was associated with an increase in the risk of depressive symptoms over time. Moderate financial hardship was associated with a greater risk of shifting from no/mild depressive symptoms to both moderate and major levels of depressive symptoms (HR = 1.42, 95% CI [1.18–1.70]; HR = 1.48, 95% CI [1.19–1.85], respectively). However, moderate financial hardship was only weakly associated with a transition from moderate to major symptoms. Severe financial hardship was systematically associated with a higher risk of progressing from no/mild to moderate (HR = 2.04, 95% CI [1.57–2.57]), from no/mild to major (HR = 2.23, 95% CI [1.66–2.98]), and from moderate to major symptoms (HR = 1.61, 95% CI [1.05–2.47]). Financial hardship was not associated with an improvement in depressive symptoms, whether it was initially moderate or severe.

**Table 4.** Hazard ratios (hrs) for transitions in depressive symptom states, EpiCov cohort (*N* = 14,236)

Transition	Moderate Financial Hardship (2) HR (_95%_ CI)	Severe Financial hardship (3) HR (_95%_CI)
Deterioration		
No/Mild (1) to Moderate (2)	**1.42 (1.18−1.70)**	**2.04 (1.57−2.67)**
No/Mild (1) to Major (3)	**1.48 (1.19−1.85)**	**2.23 (1.66−2.98)**
Moderate (2) to Major (3)	1.29 (0.87−1.90)	**1.61 (1.05−2.47)**
Improvement		
Moderate (2) to No/Mild (1)	0.97 (0.90−1.04)	0.93 (0.84−1.03)
Major (3) to No/Mild (1)	0.92 (0.76−1.10)	0.85 (0.69−1.04)
Major (3) to Moderate (2)	0.97 (0.64−1.47)	0.96 (0.59−1.58)

Multi-state model covariates included age, sex, history of psychiatric conditions, highest degree diploma, physical disability, history of somatic conditions, immigration status, receipt of benefits, household composition, and household income (baseline); perceived health status, employment status, and tobacco consumption (time-varying). HRs are reported as HR (95% CI), bold if significant with 95% confidence.

Analyses stratified by income are shown in Table 5. The association between severe financial hardship and the deterioration of depressive symptoms proved to be stronger in the highest income stratum (2.69 [1.55–4.68], 2.95 [1.73–5.00], 2.41 [1.00–5.78]) compared to the other groups (2.08 [1.24–3.48], 1.71 [0.95–3.09], 2.86 [0.98–8.33] for low income; 1.88 [1.32–2.68], 2.39 [1.62–3.53], 1.22 [0.65–2.29] for intermediate income). There was no similar trend observed for moderate financial hardship.

**Table 5.** Income-stratified hazard ratios (HRs) for transitions in depressive symptom states, EpiCov cohort (*N* = 14,236).

Income	Transition	Moderate Financial Hardship (2) HR (_95%_ CI)	Severe Financial hardship (3) HR (_95%_ CI)
**Low**	Deterioration		
	No/Mild (1) to Moderate (2)	1.46 (0.93−2.31)	**2.08 (1.24−3.48)**
	No/Mild (1) to Major (3)	1.24 (0.76−2.04)	1.71 (0.95−3.09)
	Moderate (2) to Major (3)	2.34 (0.81−6.77)	2.86 (0.98−8.33)
	Improvement		
	Moderate (2) to No/Mild (1)	0.87 (0.74−1.03)	0.85 (0.70−1.03)
	Major (3) to No/Mild (1)	0.98 (0.65−1.48)	0.91 (0.62−1.34)
	Major (3) to Moderate (2)	1.27 (0.50−3.24)	0.94 (0.38−2.36)
**Intermediate**	Deterioration		
	No/Mild (1) to Moderate (2)	**1.33 (1.03 − 1.71)**	**1.88 (1.32−2.68)**
	No/Mild (1) to Major (3)	**1.62 (1.18−2.23)**	**2.39 (1.62−3.53)**
	Moderate (2) to Major (3)	1.12 (0.65−1.92)	1.22 (0.65−2.29)
	Improvement		
	Moderate (2) to No/Mild (1)	1.01 (0.91−1.13)	0.99 (0.87−1.14)
	Major (3) to No/Mild (1)	0.88 (0.68−1.15)	0.85 (0.63−1.16)
	Major (3) to Moderate (2)	0.93 (0.52−1.67)	1.00 (0.49−2.01)
**High**	Deterioration		
	No/Mild (1) to Moderate (2)	**1.52(1.14−2.02)**	**2.69 (1.55−4.68)**
	No/Mild (1) to Major (3)	1.30 (0.89−1.88)	**2.95 (1.73−5.00)**
	Moderate (2) to Major (3)	1.03 (0.49−2.19)	**2.41 (1.00−5.78)**
	Improvement		
	Moderate (2) to No/Mild (1)	0.99 (0.86−1.13)	0.82 (0.60 − 1.12)
	Major (3) to No/Mild (1)	0.91 (0.69−1.19)	0.67 (0.44−1.02)
	Major (3) to Moderate (2)	1.01 (0.49−2.07)	0.97 (0.34−2.71)

Multi-state model covariates included age, sex, history of psychiatric conditions, highest degree diploma, physical disability, history of somatic conditions, immigration status, receipt of benefits, household composition, and household income (baseline); perceived health status, employment status, and tobacco consumption (time-varying). HRs are reported as HR (95% CI), bold if significant with 95% confidence.

## Discussion

Using data from a large nationally representative cohort from the French population, surveyed from 2020 to 2022, our work found that the experience of financial hardship was associated with subsequent depressive symptoms. This association appeared to be stronger among high-income households. Multi-state models (MSM) allowed us to study the evolution of depressive symptoms rather than their cross-sectional state. Our study could be of interest to decision-makers interested in cross-sectoral approaches to improve mental health in the general population.

### Comparison with past studies

#### Financial hardship and depression

Past research has documented a link between perceived financial hardship and mental health outcomes, especially depression (Ridley *et al.*, 2020). This association was also observed during the COVID-19 period with low or medium effect sizes (Chatterji *et al.*, 2021; Lueck, 2021). Our study yielded similar findings in a nationally representative sample of the French population. We decided to use self-reported experiences of financial hardship to take into consideration the subjective perception of financial struggles rather than their objective reality while studying the interactions between these two variables. This approach proved efficient in studying the onset and deterioration of depressive symptoms. On the other hand, subjective financial hardship was not associated with the improvement of depressive symptoms, either positively or negatively. Our results fall in line with recent research opting for subjective measures of financial hardship rather than objective ones. Our study not only supports the value of subjective measures of economic situations but also reveals that the simultaneous exploration of subjective and objective measures of financial struggles highlights interactions that are relevant to mental health. We indeed found that the relationship between perceived financial struggles and mental health was stronger in high-income households compared to intermediate or low-income households. This finding contrasts with the most common results, which state that mental health problems mostly affect lower-income populations (Ridley *et al.*, 2020).

One possible explanation is that financial hardship in high-income households is linked to short-term economic negative events, such as job loss or debt, which could be associated with depressive symptoms (Selenko and Batinic, 2011). On the other hand, financial hardship is more structural in low-income households (Kim, 2021); thus, its link with changes in mental health could be less likely. Secondly, it may be that depression levels among people who experience high levels of financial hardship are so high that they are unlikely to increase further, making the identification of transitions notably harder for the MSM. Thirdly, the perception of financial hardship could matter more than objective financial circumstances with respect to mental health outcomes. This idea was explored in research prior to the COVID-19 pandemic(Asebedo and Wilmarth, 2017) and one study briefly explored the issue during the pandemic(Sultana *et al.*, 2021). Individuals with high income could have elevated levels of spending and debt, entailing rapid changes in their income, which could lead to deterioration in their mental health. Individuals belonging to the highest income decile indeed reported high levels of deterioration in their financial situation (Bajos *et al.*, 2020). This is also consistent with research showing the mental health consequences of income instability (Pryor *et al.*, 2019). Understanding the mechanisms that link financial strain to mental health could be crucial in guiding healthcare and economic policies, as suggested by Ridley *et al.* (Ridley *et al.*, 2020). Our findings could be assessed in other countries and other settings to pursue this objective. The pandemic context of the study obviously raises the question of the generalization of the results. Several arguments are in favour of a generalization to a non-pandemic context. First, the pandemic levels of depression and financial hardship being inflated does not necessarily imply that the association between them was different at the time. Furthermore, preparedness for future epidemics is considered crucial given the global impact of COVID-19 (Williams *et al.*, 2023).

#### Multi-state models

MSM have been essentially used in previous research to model the transition from depression to somatic events, including death (Meyer *et al.*, 2019; Qiao *et al.*, 2021). We found that they were also useful for modelling transitions across depressive states over time, a topic that few studies have explored (Xiong *et al.*, 2021). Studying multiple depressive states allows to draw away from binary measures, opening the exploration of the deterioration and improvement of symptoms rather than the appearance and disappearance of psychometrically evaluated depression. MSM could then be used as an opportunity to model risk factors for depression in a clinically representative manner, providing highly interpretable and clear results. Opting for a model is not merely a choice made for statistical reasons; it is also a statement on how the process linking exposure to outcome is conceptualized. We could consider depressive states as sudden events occurring in continuous time—with the risk of occurrence being higher in a situation of perceived financial hardship. This approach coincides to some extent with psychological theories of depression, some of which state that life events can trigger the onset of depressive symptoms (Shapero *et al.*, 2014), advocating the use of MSM.

#### Strengths and limitations

Our study presents several limitations. Firstly, the participants’ income was used as a measure of their objective financial situation. Household income was extracted from the 2018 tax return, 2–4 years prior to the survey. Using tax-based data was, however, a more precise measure of the participants’ financial means than self-reported data. Secondly, the high level of missing data could have led to an underestimation of participants’ depressive symptoms, considering that depressed participants are prone to attrition, therefore tempering effect sizes. However, self-reported measures such as the PHQ9 are valid and make it possible to identify levels of depressive symptoms in general population surveys (Cameron *et al.*, 2011). Thirdly, the history of depressive disorders was not taken into account in our study. However, we took the history of severe psychiatric disorders into account, which probably captured part of the variability associated with prior experiences of depression. Finally, we did not consider the possibility of bidirectionality. This choice was made to focus on our main objective, which is to guide public policies to reduce mental health difficulties. Our study also has strengths, which we would like to highlight. Firstly, we used a large sample from nationally representative weighted data, making our estimates easily generalizable to the population of France. Secondly, we were able to check for many covariates, taking account of potential confounders reported in previous research. Thirdly, stratification on the participants’ income helped highlight interactions between perceived financial hardship and income in their association with depressive symptoms. Lastly, we used a clinical interpretation of the PHQ-9 (Kroenke *et al.*, 2001) to make sure the depressive states were as close to a realistic diagnosis as possible, unlike other studies which often applied cutoff values to the linear psychometric scales they used (Xiong *et al.*, 2021).

## Conclusion

In a French population-based cohort study, perceived financial hardship was found to be associated with later depressive symptoms, particularly among individuals belonging to the highest income stratum. Multi-state models (MSM) provided a framework that was faithful to the theoretical models of depression while providing easily interpretable insights into the evolution of depressive symptoms in cases of financial hardship. Overall, our results highlight the interaction between individuals’ socio-economic position, their perception of it, and their mental health. The importance of individuals’ perception might indicate that public policies should focus on financial stability and insecurity rather than financial status to reduce the onset and deterioration of depressive symptoms.

## Supporting information

10.1017/S2045796025100115.sm001 Maffre Maviel et al. supplementary material Maffre Maviel et al. supplementary material

## Data Availability

The EpiCov dataset is available for research purposes concerning the baseline and first and second follow-ups on CASD (https://www.casd.eu/). Any researcher may access the dataset after submitting a request to the EpiCov data operation committee (via email to the corresponding author) for approval, in accordance with the French Ethics and Regulatory Committee procedure (Comité du Secret Statistique, CESREES, and CNIL).

## Supplementary material

The supplementary material for this article can be found at https://doi.org/10.1017/S2045796025100115.
