# Supplementary material for: Experience of financial hardship and depression: a longitudinal population-based multi-state analysis
Source: Epidemiol Psychiatr Sci. 2025 Jul 1;34:e36. doi: 10.1017/S2045796025100115 (PMC12281044; doi:10.1017/S2045796025100115)
Supplement: Maffre Maviel et al. supplementary material [file S2045796025100115sup001.docx]

**Supplementary Material**

**Results for each imputation method**

Attrition was not selective for PHQ-9 (average score 4.04 among full respondents, 4.01 among incomplete respondents. On the other hand, it was slightly selective for financial hardship (40% reported hardship among full respondents, 52% among others).

Four different methods were used to handle missing data:

1. Imputation of missing covariates and exposition factor
2. Imputation of missing covariates and exposition factor combined with inverse probability weighting (IPW) (1)
3. Imputation of missing covariates, exposition and outcome
4. Imputation of missing covariates, exposition factor and outcome combined with IPW (2)

Inverse probability weights were used to account for attrition bias by balancing covariates for complete and uncomplete cases. Propensity scores were computed through a logistic regression model evaluating the probability to leave the survey mid-way through. The covariates used in the logistic regression were identical to those used in the multi-state models for adjustment. Average treatment effect (ATE) weights were used because of the similarity between both groups in terms of size and covariate distribution. Weights were estimated with R package *WeightIt* (0.13.1). All covariates were balanced after weighting, with a standardized mean difference (SMD) threshold of 0.1.

| ***Supplementary Table S1.*** *Hazard Ratios (HRs) for transitions in depressive symptoms states with imputation method 1.* | | |
| --- | --- | --- |
| **Transition** | **Moderate Financial Hardship (2)** | **Severe Financial hardship (3)** |
| Deterioration |  |  |
| No/Mild (1) to Moderate (2) | **1.52 (1.26-1.83)** | **2.42 (1.88-3.11)** |
| No/Mild (1) to Major (3) | **1.51 (1.22-1.87)** | **2.30 (1.69-3.15)** |
| Moderate (2) to Major (3) | 1.37 (0.92-2.03) | **1.76 (1.10-2.82)** |
| Improvement |  |  |
| Moderate (2) to No/Mild (1) | 0.94 (0.86-1.03) | 0.91 (0.80-1.03) |
| Major (3) to No/Mild (1) | 0.89 (0.76-1.04) | 0.94 (0.79-1.12) |
| Major (3) to Moderate (2) | 0.83 (0.54-1.26) | 0.91 (0.55-1.52) |
| Multi-state model covariates included age, sex, history of psychiatric condition, highest degree diploma, physical disability, history of somatic condition, immigration status, receipt of benefits, household composition and household income (baseline); perceived health status, employment status and tobacco consumption (time-varying). HRs are reported as HR (95% CI), bold if significant with 95% confidence. | | |

| ***Supplementary Table S2.*** *Hazard Ratios (HRs) for transitions in depressive symptoms states with imputation method 2.* | | |
| --- | --- | --- |
| **Transition** | **Moderate Financial Hardship (2)** | **Severe Financial hardship (3)** |
| Deterioration |  |  |
| No/Mild (1) to Moderate (2) | **1.46 (1.18-1.80)** | **2.22 (1.64-2.98)** |
| No/Mild (1) to Major (3) | **1.58 (1.24-2.01)** | **2.15 (1.52-3.04)** |
| Moderate (2) to Major (3) | **1.51 (1.00-2.27)** | **1.83 (1.15-2.93)** |
| Improvement |  |  |
| Moderate (2) to No/Mild (1) | 0.94 (0.86-1.03) | 0.90 (0.79-1.02) |
| Major (3) to No/Mild (1) | 0.91 (0.77-1.07) | 0.91 (0.74-1.10) |
| Major (3) to Moderate (2) | 0.78 (0.50-1.22) | 1.04 (0.63-1.71) |
| Multi-state model covariates included age, sex, history of psychiatric condition, highest degree diploma, physical disability, history of somatic condition, immigration status, receipt of benefits, household composition and household income (baseline); perceived health status, employment status and tobacco consumption (time-varying). HRs are reported as HR (95% CI), bold if significant with 95% confidence. | | |

| ***Supplementary Table S3.*** *Hazard Ratios (HRs) for transitions in depressive symptoms states with imputation method 4.* | | |
| --- | --- | --- |
| **Transition** | **Moderate Financial Hardship (2)** | **Severe Financial hardship (3)** |
| Deterioration |  |  |
| No/Mild (1) to Moderate (2) | **1.39 (1.15-1.67)** | **2.00 (1.54-2.60)** |
| No/Mild (1) to Major (3) | **1.53 (1.22-1.90)** | **2.12 (1.58-2.84)** |
| Moderate (2) to Major (3) | 1.37 (0.94-1.98) | **1.62 (1.06-2.47)** |
| Improvement |  |  |
| Moderate (2) to No/Mild (1) | 0.96 (0.89-1.04) | 0.93 (0.84-1.02) |
| Major (3) to No/Mild (1) | 0.93 (0.78-1.11) | 0.84 (0.69-1.03) |
| Major (3) to Moderate (2) | 0.92 (0.61-1.39) | 1.06 (0.67-1.67) |
| Multi-state model covariates included age, sex, history of psychiatric condition, highest degree diploma, physical disability, history of somatic condition, immigration status, receipt of benefits, household composition and household income (baseline); perceived health status, employment status and tobacco consumption (time-varying). HRs are reported as HR (95% CI), bold if significant with 95% confidence. | | |

| ***Table S4.*** *Characteristics of the study population, EpiCov cohort, at baseline/wave 1 (May 2020)* | |  |  |
| --- | --- | --- | --- |
| **Characteristic** | **No financial hardship**  **N = 8,210** | **Moderate financial hardship**  **N = 4,405** | **Severe financial hardship**  **N = 1,592** |
| **Demographic characteristics** | | | |
| Age, Mean (SD) | 48.8 (18.9) | 47.4 (17.8) | 44.5 (15.2) |
| Female, n (%) | 4,329 (52.7%) | 2,420 (54.9%) | 900 (56.5%) |
| Immigration status, n (%) |  |  |  |
| Born FR. From FR. Parents | 6,649 (83.5%) | 3,426 (80.8%) | 1,091 (72.5%) |
| Descendant of immigrant | 568 (7.1%) | 372 (8.8%) | 218 (14.5%) |
| Immigrant | 754 (9.4%) | 444 (10.5%) | 195 (13%) |
| Missing | 248 | 163 | 88 |
| Type of household, n (%) |  |  |  |
| Alone | 1,101 (13.4%) | 618 (14%) | 300 (18.9%) |
| Couple without children | 2,654 (32.4%) | 1,078 (24.5%) | 240 (15.1%) |
| Couple with at least one child | 3,347 (40.8%) | 1,853 (42.1%) | 618 (38.9%) |
| Single-parent | 476 (5.8%) | 455 (10.3%) | 253 (15.9%) |
| Other | 625 (7.6%) | 397 (9%) | 178 (11.2%) |
| Missing | 7 | 4 | 3 |
| **Measures of socio-economic position** | | | |
| Household income, n (%) |  |  |  |
| Low (1^st^-2^nd^ decile) | 783 (9.8%) | 978 (17.5%) | 652 (42.6%) |
| Medium (3^rd^-7^th^ decile) | 3,075 (38.5%) | 2,347 (55.2%) | 727 (47.5%) |
| High (8^th^-10^th^ decile) | 4,121 (51.6%) | 928 (21.8%) | 150 (9.8%) |
| Missing | 231 | 152 | 63 |
| Employment status, n (%) |  |  |  |
| Unemployed/Retired | 3,985 (48.5%) | 2,108 (47.9%) | 782 (49.1%) |
| Employed | 4,225 (51.5%) | 2,297 (52.1%) | 810 (50.9%) |
| Highest degree, n (%) |  |  |  |
| No high school degree | 1,262 (5.4%) | 983 (22.3%) | 362 (22.7%) |
| High school degree | 4,091 (49.8%) | 2,611 (59.2%) | 975 (61.2%) |
| > 2 years higher education | 2,857 (34.7%) | 811 (18.4%) | 255 (16%) |
| Receipt of benefits, n (%) | 1,120 (13.6%) | 1,153 (26.2%) | 665 (41.8%) |
| **Health characteristics** | | | |
| History of a psychiatric condition, n (%) | 32 (0.4%) | 31 (0.7%) | 19 (1.2%) |
| Missing | 0 | 1 | 0 |
| Self-reported health status, n (%) |  |  |  |
| Very Bad/Bad | 197 (2.4%) | 184 (4.2%) | 136 (8.6%) |
| Good | 8,000 (97.6%) | 4,214 (95.8%) | 1,453 (91.4%) |
| Missing | 13 | 7 | 3 |
| Physical disability, n (%) | 191 (2.3%) | 176 (4%) | 94 (5.9%) |
| Missing | 0 | 1 | 0 |
| History of somatic condition, n (%) | 2,027 (24.7%) | 1,236 (28%) | 467 (29.3%) |
| Missing | 0 | 1 | 0 |
| Tobacco consumption, n (%) |  |  |  |
| None | 6,898 (84%) | 3,312 (75.2%) | 997 (62.6%) |
| Less than once a day | 292 (3.6%) | 161 (3.7%) | 76 (4.8%) |
| At least once a day | 1,020 (12.4%) | 930 (21.1%) | 519 (32.6%) |
| Missing | 0 | 2 | 0 |

**References**

1. Seaman SR, White IR, Copas AJ, Li L. Combining Multiple Imputation and Inverse-Probability Weighting. Biometrics. mars 2012;68(1):129‑37.

2. Kang JDY, Schafer JL. Demystifying Double Robustness: A Comparison of Alternative Strategies for Estimating a Population Mean from Incomplete Data. Stat Sci. nov 2007;22(4):523‑39.
